# Ochrobactrum anthropi infection following corneal transplantation -a case report and review of literature

**DOI:** 10.1186/s12886-024-03472-z

**Published:** 2024-06-03

**Authors:** Lei Liu, Chunmei Wang, Hui Xu, Lulu Hou, Rong Huang, Xiaoru Shi, Hui Jia

**Affiliations:** Department of ophthalmology, The first hospital of Jilin University, Changchun, 130021 China

**Keywords:** Corneal transplantation, Postoperative infection, Ochrobactrum anthropi, Metagenomic next-generation sequencing

## Abstract

**Background:**

Ochrobactrum anthropi is widely distributed and primarily infects patients with compromised immune functions . Historically, O. anthropi has been considered to possess low toxicity and pathogenicity; however, recent studies suggest that it may in fact cause severe purulent infections. In this case study, we examine a case of O. anthropi infection following corneal transplantation, exploring the occurrence and outcomes of such post-operative infections.

**Case presentation:**

A retrospective analysis of cases involved examinations, genetic testing for diagnosis, and subsequent treatment. In patients undergoing partial penetrating keratoplasty with a fungal corneal ulcer perforation, anterior chamber exudation and purulence were observed post-surgery. Despite antifungal treatment, genetic testing of the anterior chamber fluid and purulent material confirmed O. anthropi infection. The use of antimicrobial treatment specifically targeting O. anthropi was found to be effective in treating the infection.

**Conclusion:**

Inflammatory reactions following corneal transplantation should be should be monitored for the presence of other infections. Genetic testing has significant implications for clinical diagnosis and treatment.

## Introduction

Ochrobactrum anthropi, a conditional pathogenic bacterium, is a Gram-negative rod that exhibits oxidase and catalase production and lacks fermentation capability. It is widely distributed and primarily infects patients with compromised immune functions [1, 2]. Reports also indicate infections in hosts without prior illnesses and with normal immune functions [3, 4]. The clinical presentation of infections caused by O. anthropi is not specific and the bacteria demonstrates robust drug resistance. This poses challenges in clinical diagnosis and treatment [5].

Historically, Ochrobactrum anthropi has been regarded as having low toxicity and pathogenicity. However, it can cause severe purulent infection [6]. Reports exist of post-organ transplantation bacteremia [7], and in ophthalmology, occurrences of intraocular inflammation following cataract surgery [8, 9]. However, cases of O. anthropi infection after corneal transplantation have not been documented. Here, we present a case of O. anthropi infection following corneal transplantation surgery.

## Case report

**Fig. 1:**
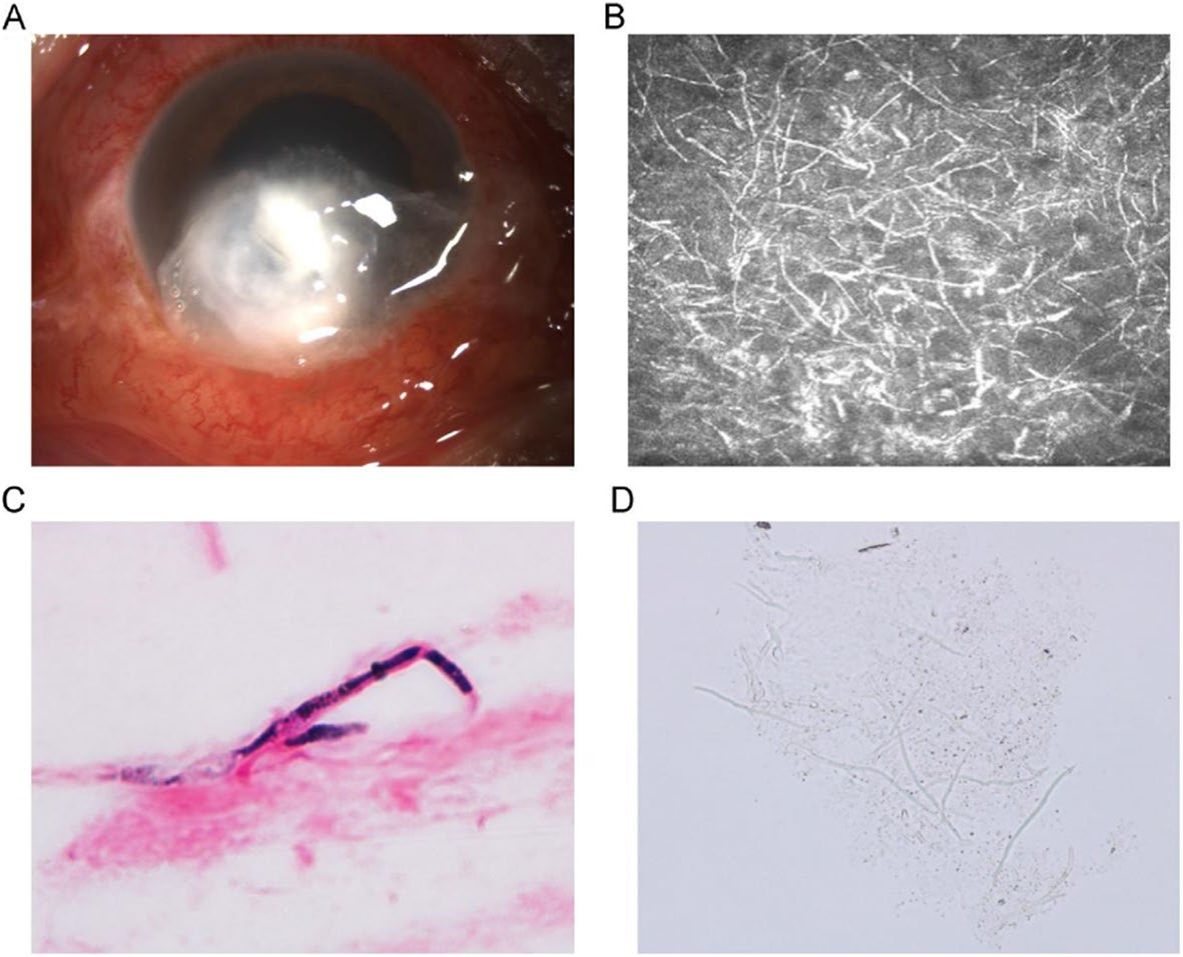
**A** The clinical presentation is characterized by peripheral central corneal opacity and infiltration, which exhibit typical elevated lesions, a dry surface, and feathery infiltrating margins. **B**. Confocal microscopy reveals the presence of fungal hyphae. **C**. Fungal hyphae are observed in corneal scrapings with Gram staining. **D**. Corneal scrapings with potassium hydroxide (KOH) wet mount demonstrate the presence of fungal hyphae

**Fig. 2:**
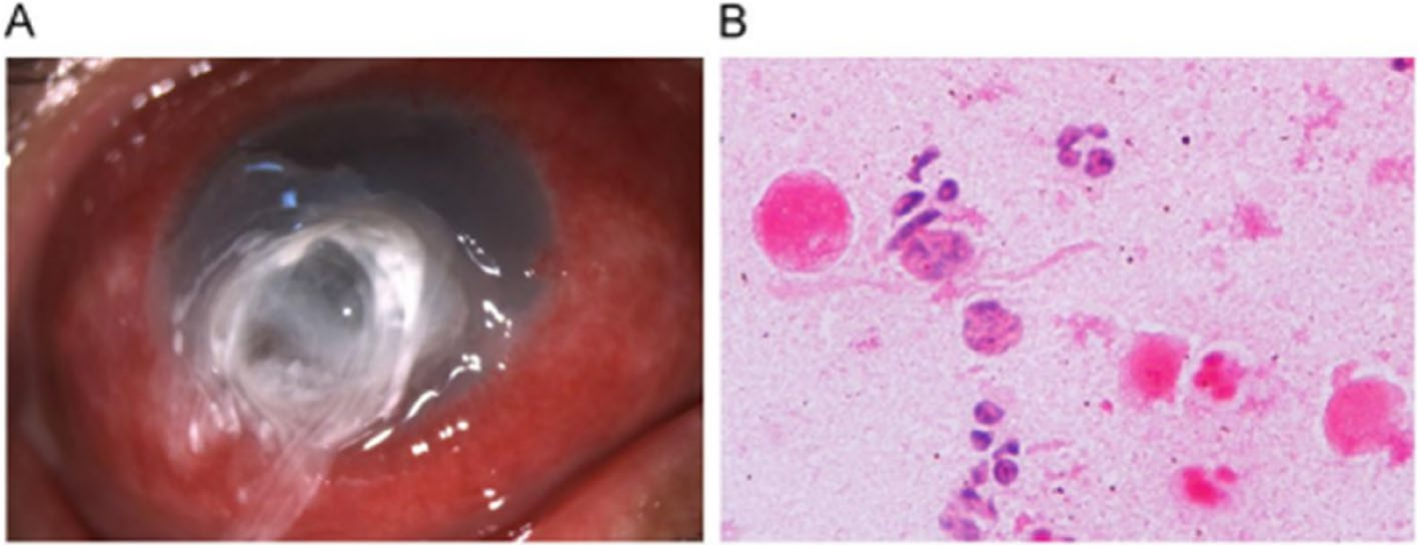
**A** After treatment, the corneal ulcer has healed, with localized margins and corneal perforation. **B** No fungal hyphae are observed in corneal scrapings with Gram staining, but inflammatory cells are present

A 51-year-old male patient presented with a 19-day history of redness and pain in the eye. The diagnosis was fungal keratitis (Fig. 1). Treatment included frequent instillation of natamycin eye drops (50mg/ml, North China Pharmaceutical, China) and intermittent application of intracorneal voriconazole injection (1mg/ml, Pfizer Limited, US). Following one month of treatment, examination results indicated that the sign of corneal ulcer healing, with no observable progress. However, there was a concerning sign of corneal perforation. (Fig. 2). B-ultrasound demonstrated the absence of abnormalities in the vitreous and retina. Consequently, a partial penetrating keratoplasty was performed with a favorable outcome. Intraoperatively, aqueous humor samples and the affected corneal tissue were collected for subsequent culture and identification of bacteria and fungi. Voriconazole injection was used throughout the procedure for corneal margin and anterior chamber irrigation. The donor cornea, sourced from our eye bank, and residual donor cornea, along with corneal preservation solution (Corneal Chamber, Alchimial, Italy), were sent for bacterial and fungal culture postoperatively. The postoperative regimen consisted of local administration of natamycin and tacrolimus eye drops (0.1%, Senju Pharmaceutical Co, Japan), as well as levofloxacin eye drops (0.5%, Senju Pharmaceutical Co, Japan). Additionally, itraconazole capsules (200 mg daily, Xi'an Yangsen Pharmaceutical Co., Ltd., China) were orally administered.

**Fig. 3:**
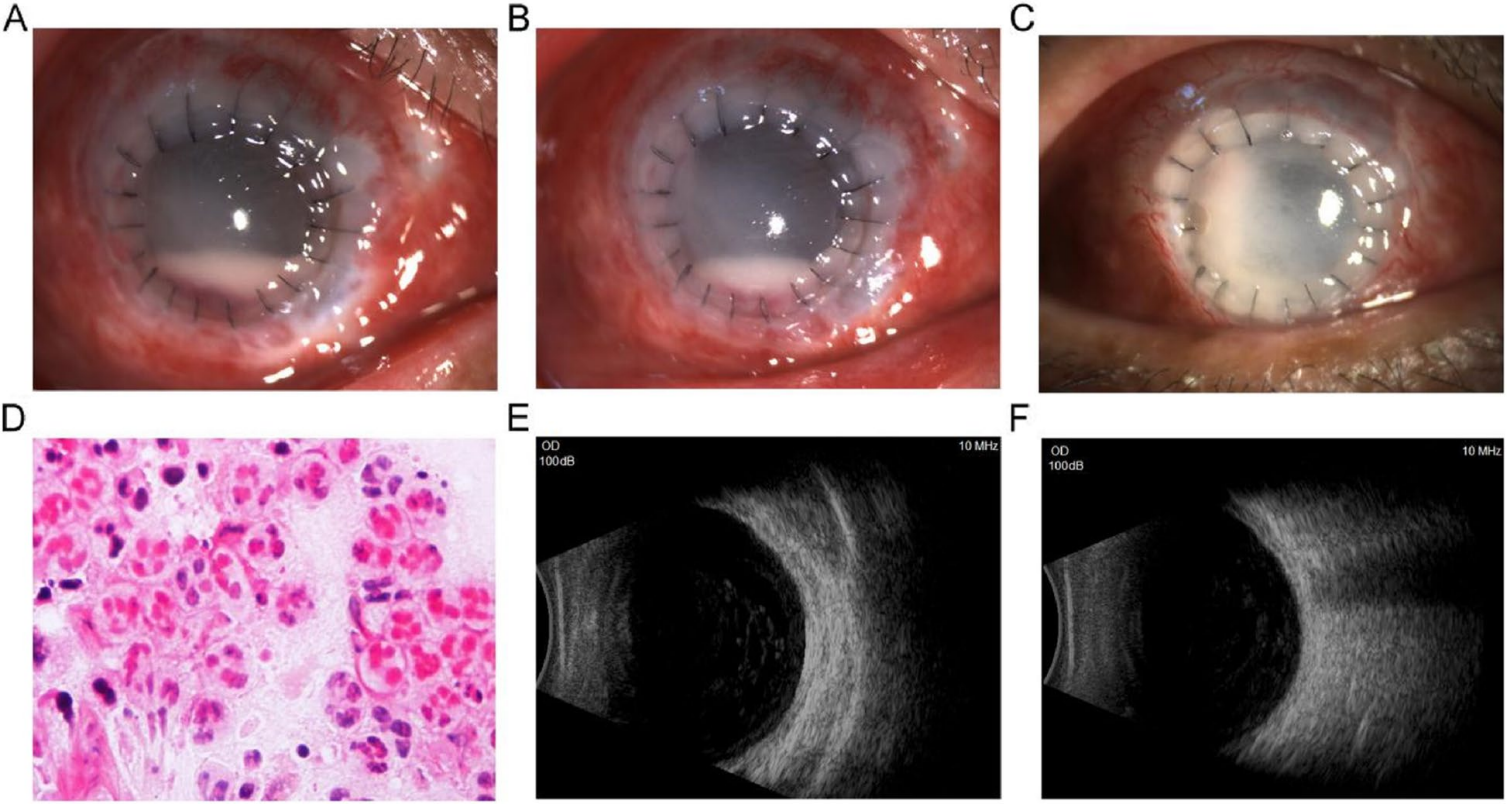
**A** Following corneal transplantation, there was an accumulation of pus in the anterior chamber of the eye, exhibiting a thin and watery consistency. **B** Subsequent to treatment, there was a discernible reduction in anterior chamber pus. **C** The corneal graft appeared grayish-white and opaque, and the anterior chamber pus completely disappeared. **D** Gram staining of corneal scrapings did not reveal any fungal hyphae, but did indicate the presence of a considerable number of inflammatory cells. **E** Turbidity was observed in the vitreous cavity, resembling sediment. **F** The vitreous opacities exhibited a noticeable reduction in comparison to the pre-treatment state

Following corneal transplantation, the graft remained transparent. However, the aqueous humor exhibited gradual turbidity, and by the fourth postoperative day, anterior chamber pus accumulation was observed, prompting concern regarding a potential recurrence of fungal infection. Consequently, the anterior chamber was aspirated to remove the pus, and a voriconazole injection was administered for anterior chamber irrigation and medication. Intraoperatively, it was noted that the anterior chamber pus was thin and watery in consistency, in contrast to the thicker purulent fluid observed in fungal infections. An examination of the anterior chamber pus revealed the absence of fungal hyphae (Fig. 3D). Postoperatively, there was no significant improvement in the anterior chamber reaction, and the cornea exhibited mild edema.

A microbiological examination of the recipient corneal ulcer, aqueous humour, donor cornea, and preservation solution yielded negative results for bacteria and fungi, which is thought to be the consequence of preoperative antifungal and antibacterial treatment. However, given the possibility of other infections occurring postoperatively, aqueous humour and anterior chamber pus from the operated eye were collected for metagenomic next-generation sequencing (mNGS) test. Total genomic DNA was extracted using a nucleic acid extraction kit, then fragmented to 200 bp and used to build a library with end-repaired, adapter-ligation, and polymerase chain reaction amplification. The prepared library was sequenced on the MGISEQ-2000 sequencing platform (MGI, China). The reads were finally aligned to the Microbial Genome Databases, which contain the whole-genome sequences of 8,472 viruses, 10,537 bacteria, 903 fungi and 288 parasites. During the aforementioned interval, the previously administered eye drops were maintained. This was accompanied by an increase in the rate of anterior chamber pus leakage, culminating in the clouding of the corneal graft (Figure 3A). The mNGS results identified O. anthropi, with 321 sequences with a relative abundance of 98.98%. Additionally, a vitreous opacity was revealed through a B-ultrasound examination. (Fig. 3E). To treat O. anthropic, ceftazidime (25 mg/ml), an amikacin (4 mg/ml), and vancomycin (10 mg/ml) were administered to the anterior chamber and vitreous, respectively, three times each. The oral antifungal medication was discontinued, and a cefuroxime injection (1.0 g, administered three times a day) was administered intracamerally. To prevent a recurrence of fungal infection, natamycin eye drops were administered four times daily, in conjunction with tobramycin eye drops (0.3%, Tobramycin Eye Drops, Alcon-Couvreur n. v, Belgium), and levofloxacin eye drops (0.5%, Senju Pharmaceutical Co, Japan) at 1-hour intervals. Following three days of treatment, there was a reduction in anterior chamber pus (see Fig. 3B). After a further ten days of treatment, the anterior chamber pus had been absorbed completely. However, the corneal graft remained cloudy (Fig. 3C), with ultrasound indicating that the vitreous opacities had reduced (Fig. 3F). The patient's condition remained stable, with effective infection control being maintained.

**Fig. 4:**
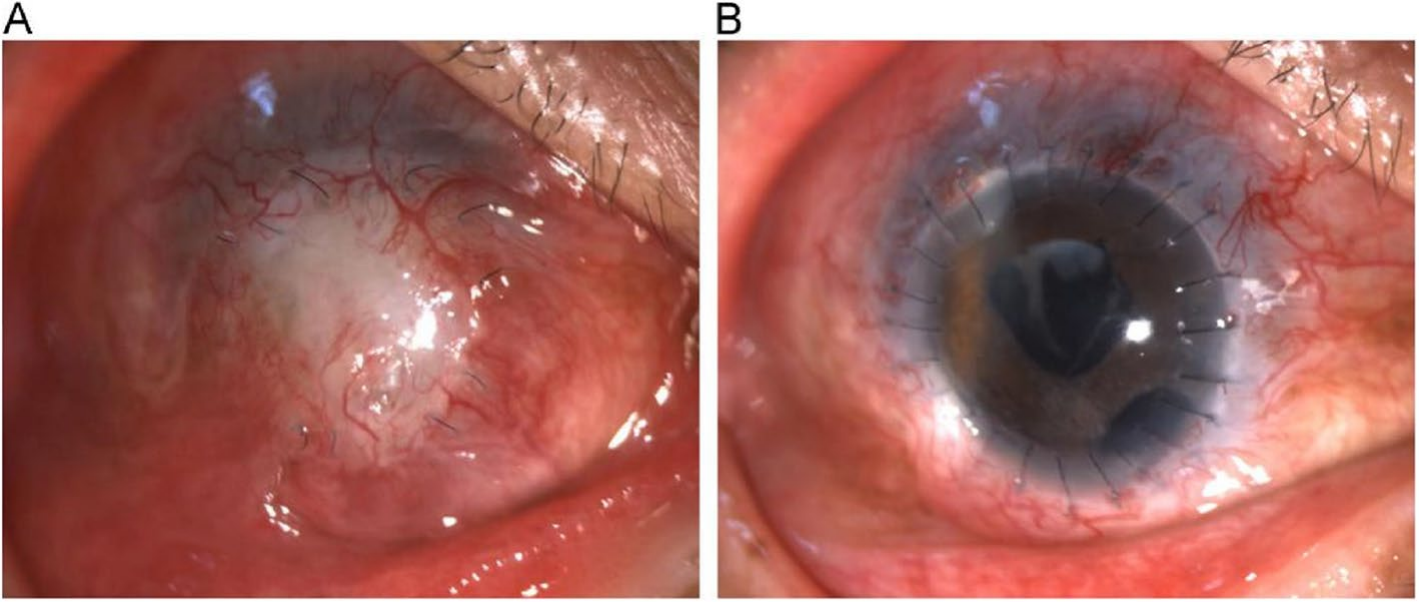
**A** Pseudo-pterygium and corneal opacities developed six months after corneal transplantation. **B** After the second corneal transplantation, the corneal graft became transparent

Six months following corneal transplantation, the operated eye exhibited corneal opacities and pseudo-pterygium (Fig. 4A). B-ultrasound examination did not reveal any abnormalities. To enhance visual function, a secondary partial penetrating keratoplasty was conducted concurrently with a cataract removal procedure. Postoperatively, Tobramycin eye drops were administered for the purpose of prophylaxis against infection, in conjunction with Tacrolimus and Prednisolone Acetate Ophthalmic Suspension (1%, Allergan Pharmaceuticals Ireland) for the prevention of rejection. One month following the procedure, the corneal graft was observed to be transparent, with a corrected visual acuity of 0.1 (Fig. 4B).

## Discussion

Fungal keratitis stands out as the predominant cause of blindness in patients with infectious corneal diseases in China [10]. Therapeutic keratoplasty serves as a pivotal intervention in the management of progressive fungal keratitis when conventional pharmaceutical therapies prove ineffective. However, a considerable contributor to the failure of corneal transplantation is the recurrence of postoperative fungal infections [11]. Risk factors for infection recurrence encompass the size of corneal ulcer infiltration, graft dimensions, and the type of fungus involved [12]. Furthermore, comprehensive antifungal treatment prior to corneal transplantation constitutes a critical determinant for surgical success. In the presented case, the patient received standardized antifungal treatment post-diagnosis, resulting in favorable therapeutic outcomes. Undertaking corneal transplantation at this juncture successfully averted postoperative recurrence of fungal infections. Hence, judiciously selecting the timing for corneal transplantation emerges as a pivotal strategy in controlling the recurrence of fungal corneal ulcers.

Anterior chamber pus was observed on the fourth day after the surgery. Given past experience, a recurrence of fungal infection was suspected and voriconazole anterior chamber lavage was performed on the operative eye. However, the observed pus appeared thin and lacked the characteristic viscosity typically seen in fungal infections [13]. Subsequent exacerbation of the patient's condition after antifungal treatment led to concerns regarding potential infection by other pathogens. In order to rule out the possibility of contamination from the donor cornea, another patient that had received a corneal transplant from the same donor at the same time was continuously monitored. No signs of infection were found.

Due to the fact that microbial cultures and smear examinations of the affected cornea failed to detect any pathogens, we conducted next-generation sequencing (NGS) on the anterior chamber pus to identify the infectious agent. Metagenomic is a diagnostic technique widely used in microbiological research but infrequently employed in routine clinical microbiological diagnostics. It offers a rapid and accurate advantage in determining the nature and type of infection in clinical diagnosis [14], NGS and shotgun sequencing particularly demonstrating excellent diagnostic value for infections on the ocular surface and within the eye [15, 16]. In the event that the traditional microbiological tests employed in ophthalmology prove to be ineffective, it can serve as a highly effective supplementary diagnostic tool, markedly enhancing the detection rate of microorganisms that are particularly challenging to cultivate in clinical samples [17].

O. anthropi is widely present in the environment, soil, and water sources (physiological saline, preservative solutions, dialysis fluid) [5, 18]. Additionally, it has been isolated from human bodily fluids [19]. As a rare conditional pathogen, most reported cases involve hospital-acquired infections, with infected patients using various indwelling and invasive medical devices such as central venous catheters, artificial heart valves, and drainage tubes [6, 20, 21]. Existing clinical case reports have found that O. anthropi-induced endophthalmitis is more common after cataract intraocular lens implantation [8, 9, 22, 23], and there is also a reported case of infection after artificial corneal implantation [24]. Infections related to implants may be associated with the propensity of O. anthropi to adhere to the surfaces of synthetic materials [25]. Some studies also suggest a correlation between post-cataract surgery endophthalmitis and contamination of intraocular irrigation fluids [22]. Another report found that after thorough cleaning of the cannula kit of an ultrasonic emulsification machine, the hospital's outbreak of O. anthropi infection promptly disappeared [26]. This report further confirms this hypothesis.

O. anthropi, being an opportunistic pathogen, manifests infection significantly in individuals with compromised immune function and a history of both local and systemic antibiotic usage [8] [27, 28]. However, in this particular case, the patient did not employ any indwelling or invasive medical devices, and comprehensive examinations, both physical and systemic, failed to reveal any symptoms. Based on the patient's medical history, the human Ochrobactrum anthropi infection in this case can be attributed to local immune function decline after corneal transplantation. In addition, the patient had a history of topical antibiotic use for 1 month prior to surgery, which increased the risk of human O. anthropi infection due to dysbiosis and antibiotic resistance.

Despite the generally perceived low virulence of O.anthropi, its infection rates have gradually increased in recent years due to its inherent multidrug resistance to antibiotics. O. anthropi exhibits resistance to β-lactam drugs, particularly cephalosporins and penicillins, while being sensitive to quinolone drugs and aminoglycoside drugs, especially amikacin and gentamicin [29, 30]. Our patient received prompt and adequate antibiotic treatment upon confirmation of the infection, effectively controlling the infection rapidly. However, it is noteworthy that, after 14 days of treatment, although the infection was well controlled, the corneal graft still exhibited cloudiness. Currently, while there have been numerous reports on endophthalmitis caused by O. anthropi, there is limited documentation on the toxicity of this bacterium to the cornea itself and the resulting pathological changes. Nandini et al. reported a case of corneal inflammation caused by O. anthropi infection, presenting as anterior chamber purulence in a patient with a history of viral keratitis. This case was ultimately diagnosed as O. anthropi infection, with corneal histopathological findings showing detachment of the posterior elastic layer [31]. Generally, bacterial infections can lead to pathological changes such as corneal opacification, edema, ulcer formation, and endothelial damage [32]. Nevertheless, in the case of corneal tissue infection by O. anthropi, which is generally regarded as a Gram-negative bacterium of low virulence, the potential exists for the pathogen to elicit more severe pathological alterations. The cloudiness of the corneal graft subsequent to O. anthropi infection in this instance may be attributed to the local eye drops and anterior chamber injections employed during the infection treatment, which may have resulted in corneal endothelial damage [33–35].

## Conclusion

The case presented constitutes the initial report of O. anthropi-induced keratitis following corneal transplantation. The clinical manifestations of the infection are lacking in specificity. Therefore, ophthalmologists must select the most appropriate diagnostic approach to identify the pathogen and guide therapeutic interventions. For patients with conditions predisposing to local immune dysfunction, the possibility of O. anthropi infection should be considered promptly upon the onset of infection. The corneal pathology induced by this pathogen warrants attention and merits further investigation.

## Data Availability

No datasets were generated or analysed during the current study.
